# Impact of the Uremic Milieu on the Osteogenic Potential of Mesenchymal Stem Cells

**DOI:** 10.1371/journal.pone.0116468

**Published:** 2015-01-30

**Authors:** Diana Lanza, Alessandra F. Perna, Adriana Oliva, Raymond Vanholder, Anneleen Pletinck, Salvatore Guastafierro, Annarita Di Nunzio, Carmela Vigorito, Giovambattista Capasso, Vera Jankowski, Joachim Jankowski, Diego Ingrosso

**Affiliations:** 1 First Division of Nephrology, Department of Cardio-thoracic and Respiratory Sciences, Second University of Naples, School of Medicine, Napoli, Italy; 2 Department of Biochemistry, Biophysics and General Pathology, Second University of Naples, School of Medicine, Napoli, Italy; 3 Nephrology Section Department of Internal Medicine, Ghent University Hospital, Ghent, Belgium; 4 Division of Medical Oncology and Hematology, Department of Clinical and Experimental Medicine, Second University of Naples, School of Medicine, Napoli, Italy; 5 Institute of Molecular Cardiovascular Research (IMCAR), RWTH Aachen University, Aachen, Germany; University of Catania, ITALY

## Abstract

Human mesenchymal stem cells (hMSCs), the precursors of osteoblasts during osteogenesis, play a role in the balance of bone formation and resorption, but their functioning in uremia has not been well defined. To study the effects of the uremic milieu on osteogenic properties, we applied an in vitro assay culturing hMSCs in osteogenic medium supplemented with serum from healthy donors and from uremic patients on hemodialysis. Compared to control, serum from uremic patients induces, in hMSC cultures, a modification of several key regulators of bone remodeling, in particular a reduction of the ratio Receptor Activator of Nuclear factor Kappa B Receptor (RANKL) over osteoprotegerin, indicating an adaptive response of the system to favor osteogenesis over osteoclastosis. However, the levels of osteopontin, osteocalcin, and collagen type I, are increased in cell medium, while BMP-2, and alizarin red staining were decreased, pointing to a reduction of bone formation favoring resorption. Selected uremic toxins, such as p-cresylsulfate, p-cresylglucuronide, parathyroid hormone, indoxyl sulfate, asymmetric dimethylarginine, homocysteine, were able to mimic some of the effects of whole serum from uremic patients. Serum from cinacalcet-treated patients antagonizes these effects. Hydrogen sulfide (H2S) donors as well as hemodialysis treatment are able to induce beneficial effects. In conclusion, bone modifications in uremia are influenced by the capability of the uremic milieu to alter hMSC osteogenic differentiation. Cinacalcet, H2S donors and a hemodialysis session can ameliorate the hampered calcium deposition.

## Introduction

In chronic kidney disease (CKD) and especially in patients on hemodialysis, chronic kidney disease-mineral and bone disorder (CKD-MBD) frequently affects quality of life, morbidity and mortality [1]. These alterations are to a large extent induced by the determinants of parathyroid hormone (PTH) secretion [2,3], and are also influenced by uremic retention solutes, especially p-cresylsulfate (pCS) and p-cresylglucuronide (pCG), indoxyl sulfate, asymmetric dimethylarginine (ADMA), and homocysteine [4–10].

Bone marrow-derived stem cells are either hematopoietic or non-hematopoietic (mesenchymal). Human bone marrow-derived mesenchymal stem cells (hMSCs) (marrow stromal cells) [11] can differentiate into osteoblasts, as well as chondrocytes, adipocytes and other cell types [12–14].

**Figure 1:**
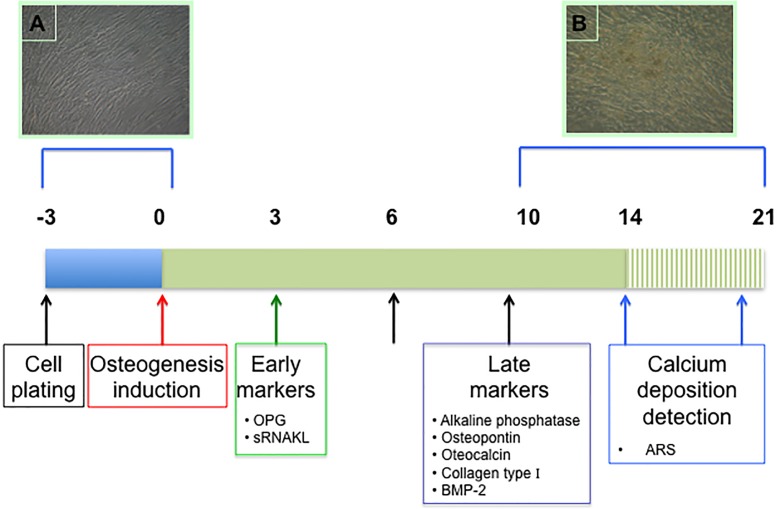
Flow chart of hMSCs culture set up and osteogenic induction. The horizontal line indicates the time period from cell plating until the beginning of calcification. Dashed bar indicates the detection of calcium deposition (ARS assay). Arrows indicate medium change with replacement of fresh serum from uremic patients or from healthy control donors. Boxes highlight specific marker detection or treatments. Early markers: OPG, sRANKL. Late markers: alkaline phosphatase, osteopontin, osteocalcin, collagen type I, BMP-2. Calcium deposition was detected by ARS assay. Differential morphology of hMSC before (fibroblast-like) and at the end of osteogenic differentiation process (spherical cells with calcium deposition) is also shown (panels A and B, respectively).

During hMSCs osteoblastic differentiation, several molecules, such as osteoprotegerin (OPG), receptor activator of nuclear factor kappa B ligand (RANKL), osteopontin, osteocalcin, collagen type I, bone morphogenic protein-2 (BMP-2), and alkaline phosphatase play a key role in this complex process, resulting in matrix formation and calcium deposition (Fig. 1).

OPG is a soluble cytokine belonging to the Tumor Necrosis Factor receptor superfamily acting as negative regulator of RANKL, which instead induces osteoclast differentiation in bone marrow through its receptor RANK. OPG acts as a decoy receptor that blocks RANKL, thus preventing RANK activation, osteoclast differentiation and bone resorption. RANKL is also produced by osteoblasts. The ratio between RANKL and OPG is considered to be a key index in bone formation/resorption: in case of high RANKL/OPG concentration ratio, osteoclastosis is favored, and when the opposite is present, osteoblastogenesis is preferred [15]. In hemodialysis patients, serum OPG concentration is higher, and serum RANKL concentration is lower than controls [16,17].

Osteopontin is an acidic phosphoprotein of bone, and produced by fully differentiated osteoblasts, which is involved in regulation of mineralization by acting as an inhibitor of apatite crystal growth, as well as promoting osteoclast function [18,19]. Serum osteopontin concentration is increased in hemodialysis patients [20,21].

Osteocalcin is a protein, which, along with collagen type I, is produced by osteoblasts and together combine extracellularly to form osteoid, the organic substrate upon which mineralization occurs [22]. In CKD-MBD, both markers are correlated with serum PTH levels [23].

BMP-2 is member of the Transforming Growth Factor-β (TGF-β) superfamily, which is able to induce bone formation [24]. In CKD-MBD, serum levels are significantly higher [25,26].

The serum concentration of bone-specific alkaline phosphatase reflects the cellular activity of osteoblasts, and is a useful marker of bone formation, also in CKD-MBD [27].

Finally, alizarin red staining [28–31] is a measure of *in vitro* calcium deposition on osteoid, thus representing the final step in bone formation.

In this study, we investigated the effects of serum from uremic patients on hemodialysis on the osteogenic differentiation of hMSCs, which are still largely unknown, through the analysis of the various markers. An *in vitro* assay system in which hMSCs cultured with osteogenic medium supplemented with healthy or uremic patient serum was applied. The effect of selected uremic solutes were tested, in particular pCS, pCG, PTH, indoxyl sulfate, ADMA, homocysteine, on the above osteogenic differentiation markers.

The effects of the calcimimetic cinacalcet, a drug utilized in the therapy of CKD-MBD, as well as the effects of hydrogen sulfide (H_2_S) donors were also investigated. H_2_S, an endogenous vasodilator and antioxidant, which concentration is reduced in uremia [32], is involved in osteoblast proliferation through enhancement of the transcriptional levels of alkaline phosphatase, osteocalcin, and collagen [33], as well as OPG/RANKL [34].

## Methods

### 1 Products and reagents

All cell culture materials were purchased from Gibco (Life Technology, Carlsbad CA, USA), and all chemicals, as well as PTH, indoxyl sulfate, ADMA, L-homocysteine and Diallyl disulfide (DADS) were from Sigma Chemical Co. (St. Louis, MO, USA), when not otherwise specified. Diallyl trisulfide (DATS) was from Cayman Chemical Co. (La Jolla, Ca, U.S.A.). PrestoBlue Cell Viability Reagent was purchased from Invitrogen (Life Technology, Carlsbad, CA, USA). pCS and pCG were synthesized at the organic chemistry department of the Ghent University, Belgium. pCS was synthesized according to Feigenbaum and Neuberg as a potassium salt [35] and pCG was synthesized and purified based on the work of Van der Eycken *et al* [36]. All reagents used were of analytical grade. Vacutainer tubes were purchased from Becton Dickinson Italia S.p.A.

### 2 Preparation of hMSCs

The bone marrow aspirate samples were obtained from the right or left posterior iliac crest of 17 patients (9 males and 8 females, aged >18 years) diagnosed with Non-Hodgkin Lymphoma without bone marrow involvement and with normal renal function. The bone marrow aspiration was performed before core biopsy using the same bone marrow core biopsy needle (Biopsybell, Modena, Italy). Written consent was obtained from all patients, and the study received approval from the institutional ethics committee of the Second University of Naples (Protocol No. 283 of 12 July 2013).

HMSC cultures were initiated as described previously [37]. Briefly, the heparinized bone marrow sample was diluted 1:5 with complete culture medium (Opti-MEM containing 10% fetal calf serum, 100 units/ml penicillin, 100 μg/ml streptomycin and 50 μg/ml sodium ascorbate), and incubated at 37°C in a 5% CO_2_ humidified atmosphere. Although present in bone marrow in an extremely low percentage in proportion to total mononuclear cells, hMSCs can be easily isolated on the basis of their ability to adhere to polystyrene plates, while cells of the hematopoietic lineage remain in suspension and can be removed.

After 48h, medium containing all non-adherent cells was centrifuged for 10min at 1000 rpm; the pellet was discarded, while the supernatant was added to the culture dish, in a 1:1 ratio with the fresh medium. In 3–4 days, several *foci* of adherent spindle-like cells appeared and reached sub-confluence in 1–2 weeks. The medium was refreshed every 3 days, each time leaving one half of conditioned medium. The cells harvested from each donor were pooled. Afterwards, cells were trypsinized, and their phenotype confirmed by flow cytometry using a wide panel of labeled monoclonal antibodies, as previously reported [38]. Cultures between the second and fourth passage were used in the experiments.

### 3 Serum samples

A control group of healthy donors and a group of uremic patients on hemodialysis (hemodiafiltration, HDF) were selected. All patients were clinically stable and recruited according to the following inclusion criteria:

- no clinical evidence of diabetes, lupus erythematosus, viral hepatitis, cancer;
- maintenance HDF thrice weekly for at least 3 months; polysulfone filters; Kt/V > 1.4.

Some of the HDF patients were treated with cinacalcet (Mimpara, Amgen Dompè, Italy). All previous transplant recipients were excluded.

Blood samples were drawn under fasting conditions and collected in the appropriate Vacutainer tubes for serum. In the patient group, this occurred immediately before the hemodialysis session (mid-week session), and before and after the hemodialysis session (for the experiments assessing the effects of hemodialysis). After blood withdrawal, the sample was centrifuged at 4°C for 15 min at 3000 rpm, serum was collected under sterile laminar flow hood, and stored at-20°C. One aliquot was immediately processed for routine biochemical analysis.

In Table 1, concentrations in control and patient serum of creatinine, urea, calcium, phosphorus, intact PTH (iPTH), and 25-OH vitamin D are depicted.

**Table 1:** Biochemical parameters of controls’ and patients’ serum

	**CONTROLS**	**PATIENTS**
**Creatinine (mg/dL)**	0.80 (0.20)	8.56 (0.74)***
**Urea (mg/dL)**	30.20 (10.00)	104.60 (11.00)**
**Calcium (mg/dL)**	9.25 (0.65)	8.72 (0.25)
**Phosphorus (mg/dL)**	3.60 (0.90)	5.13 (0.29)*
**iPTH (pg/mL)**	35.00 (5.20)	326.20 (35.93)***
**Vit. D 25-OH (ng/mL)**	25.60 (5.10)	8.15 (3.15)**

Serum concentrations of creatinine, urea, calcium, phosphorus, iPTH, and 25-OH vitamin D were measured in healthy controls and uremic patients, respectively. Values were expressed as mean (SE).

*p<0.05

**p≤0.01

***p<0.001.

Prior to each experiment, the required amount of serum from healthy controls (control serum) or serum from uremic patients on hemodialysis (uremic serum) was obtained by respectively pooling together all the various aliquots stored as above, using always the same pool.

### 4 Cell treatments

The ability of isolated hMSCs to grow in the presence of human serum was tested in preliminary experiments, in order to optimize growth and viability, and these conditions were used in experimental set-up. We initially checked the ability of hMSC to grow in medium supplemented with human serum at different percentage, compared to 10% fetal calf serum as a standard cell culture condition [38]. We detected optimal cell growth using 10% human serum for 3 days, which insured excellent cell viability. Therefore in all our experiments we utilized 10% human serum.

Isolated hMSCs were induced to differentiate into osteoblasts following a standard protocol [38–41]. Cells were seeded at a density of 10^4^ cells/cm^2^ in 6-well plates and the following day stimulated with either standard growth (non-osteogenic) medium (Opti-MEM) or osteogenic medium (Opti-MEM, plus 0.1 μM dexamethasone, 10 mM sodium β-glycerophosphate, 0.05 mM L-Ascorbic acid and 0.5 mM CaCl_2_) for 15–21 days. Normal or osteogenic medium were supplemented with 10% control serum, or 10% uremic serum pools, or with 10% control serum plus toxins, at final concentrations comparable to those present in uremia [42–44], i.e.: pCS and pCG at 0.48 mM and 80 μM, respectively; PTH 106 pM (1000 pg/ml); indoxyl sulfate (80 μM); ADMA (1 μM); homocysteine (100 μM). Where indicated, uremic serum was added with DADS or DATS (organosulfur H_2_S donors derived from garlic), at 200 μM final concentration [45–47]. About 30% of uremic toxins are protein-bound in circulation [48], while albumin binding capacity (its concentration in the serum being 530–770 μmol/L) highly exceeds *in vivo*, by orders of magnitude, the concentrations of most of these protein-bound retention solutes. Therefore, we set the experimental conditions so that albumin never became limiting for protein-bound retention solutes utilized. Medium was refreshed every 2–3 days and an aliquot of the supernatant was saved for determination of the relevant dosages (Fig. 1).

An adequate number of culture wells was prepared in order to have, for each set of experiments a sufficient number of parallel samples to test all the indicated markers during differentiation, as indicated in Fig. 1.

Hemofiltration is a hemodialysis technique based on uremic toxin removal obtained through convection. Hemofiltrates from 3 patients were pooled and concentrated by reversed-phase chromatography in the presence of a cationic ion-pair reagent (triethylammonium acetate (TEAA) 40 mM) [49]. Retained substances were eluted by using increasing concentrations of ethanol in water: 20%, 40%, 60%, 80% and 100% (20%= 20% ethanol in water, etc.), [49]. Therefore, the first fraction, obtained with 20% ethanol, contains a higher concentration of hydrophilic uremic toxins, and this concentration decreases with the ethanol percentage increase. The hemofiltrates were dried in SpeedVac Concentrator (Thermo Scientific, Thermo Fisher Scientific Inc.—Boston, MA, USA) to remove ethanol, and resuspended in absolute medium (D-MEM). Various dilutions of the hemofiltrate pool fractions were tested on MSC viability and osteogenic differentiation.

### 5 Cell viability and osteogenic differentiation markers

#### 5.1 Alkaline Phosphatase and Alizarin Red Staining (ARS)

After 10 days, alkaline phosphatase specific activity was evaluated. Once the medium was removed, wells were rinsed with 20 mM Tris/HCl, pH 7.4, 0.5 M NaCl (TBS) and the cells lysed by a specific buffer (20 mM Tris/HCl, pH 7.4, 0.5 mM NaCl, 0.25% Triton X-100, 0.5 mM phenylmethylsulfonyl fluoride, 0.5 mM DTT). After 30 min on ice, cell lysates were centrifuged at 13000xg for 5 min. Alkaline phosphatase activity was determined on the supernatant by measuring the release of *p*-nitrophenol from disodium *p*-nitrophenyl phosphate. The reaction mixture contained 10 mM disodium *p*-nitrophenyl phosphate, 0.5 mM MgCl_2_, 0.1 M diethanolamine phosphate buffer pH 10.5, and 10–30 mg of cell lysate in a final volume of 500 μl. After 30 min at 37°C, the reaction was stopped by adding 500 μl of 0.5 M NaOH; *p*-nitrophenol levels were measured with spectrophotometer at 405 nm wavelength. One unit was defined as the amount of enzyme that hydrolyzes 1 nmol of *p*-nitrophenyl phosphate x min^-1^. Specific activity is expressed as nmol/mg x min and is corrected for protein concentration of the cell lysates.

ARS, an anthraquinone derivative, may be used to identify calcium deposition in cell cultures. After 15–21 days of differentiation, cells were fixed with 4% paraformaldehyde for 15–30 min, washed with bidistilled water and stained with ARS at 40 mM, pH 4.1. After 20 min incubation at room temperature with gentle shaking, the unincorporated dye was removed and cells washed with bidistilled water. Calcium deposits were visible as a red staining. For quantification, 800 μl 10% acetic acid was added to each well. Cell monolayers were then scraped and transferred to a 1.5 ml microcentrifuge tube. After vortexing for 30 s, the tube was heated at 85°C for 10 min, transferred on ice for 5 min, and centrifuged at 20000xg for 15 min. Then, 500 μl of the supernatant were removed and transferred to a new tube, and 200 μl of 10% ammonium hydroxide were added to neutralize the acid. Aliquots (150 μl) of the supernatant were read in triplicate with a spectrophotometer set at 405 nm wavelength.

#### 5.2 PrestoBlue viability assay

PrestoBlue is a resazurin-based solution that functions as a cell viability indicator by using the reducing power of living cells, to quantitatively measure cell proliferation. When added to cells, the PrestoBlue reagent is modified by the reducing environment of the viable cell and turns red, becoming highly fluorescent. Viable cells retain the ability to reduce resazurin into resorufin. Nonviable cells rapidly lose metabolic capacity and thus do not generate a fluorescent signal. This colour is detected using fluorescence or absorbance measurements. Cells were washed with PBS, incubated ≥10min at 37ºC with 500 μl PrestoBlue 1X (in D-MEM without phenol red); then, for each sample, the reagent was removed and saved in a tube, cells were washed 2 times with 1 ml PBS and the supernatant saved in the same tube and then fluorescence was read at 560 nm wavelength [50,51]. The advantage of this assay is that it allows continuous monitoring of cultures, as the culturing process continued after the screening test.

#### 5.3 ELISA

Enzyme concentrations in the treated hMSCs culture medium were determined utilizing the relevant ELISA kits according to the supplier’s protocols. ELISA Kits were: Human Soluble Receptor Activator of Nuclear Factor-KB Ligand (sRANKL Total), human osteocalcin and human Osteoprotegerin (OPG), (BioVendor—BioVendor-Laboratorni medicina a.s., Brno, Czech Republic), Human osteopontin and Human Bone morphogenic protein-2 (BMP-2) (Biorbyt Ltd., Cambridge, UK), Human collagen type I (Uscn Life Science, Houston, USA).

As illustrated in Fig. 1, for the OPG and sRANKL assays (early markers), culture media obtained after 3 days of differentiation were used. For the assays of osteopontin, BMP-2, collagen type I and osteocalcin (late markers), media after 10 days were used.

### 6 Statistical Analysis

Data are presented as mean (SE). All experiments were done at least in triplicate except when otherwise stated. Within the same assay session, each sample of a set relevant to individual treatments was run in triplicate. Results on the levels of each marker were reported in the relevant units (in the experiments concerning evaluation of uremic serum effects vs. control serum). Considering experiments using uremic toxins, the difference between the measured effects of each toxin vs. control serum was reported as percentage. A paired t Student test was performed [52]. All calculations were performed using the software package GraphPad Prism, Version 5.0 for Windows (GraphPad Software, San Diego, CA, USA). Statistical significance is considered at p<0.05. Grubbs’ method was used to assess outliers.

## Results

### 1 Effects of various treatments on cells viability

We preliminarily checked cell viability under the various cell growth conditions and treatment that we employed, using the Presto Blue test assay performed both at 3 and 10 days of culture. Treatments with serum from healthy donors (control serum) and with serum from uremic patients on hemodialysis (uremic serum) and/or osteogenic medium as well as the addition of uremic toxins to medium did not significantly compromised cell viability compared to standard growth conditions (data not shown); see also ref. [37–41]. Therefore, we could rule out the occurrence of any acute or sub-acute toxic effect of added compounds in our experimental conditions.

**Figure 2:**
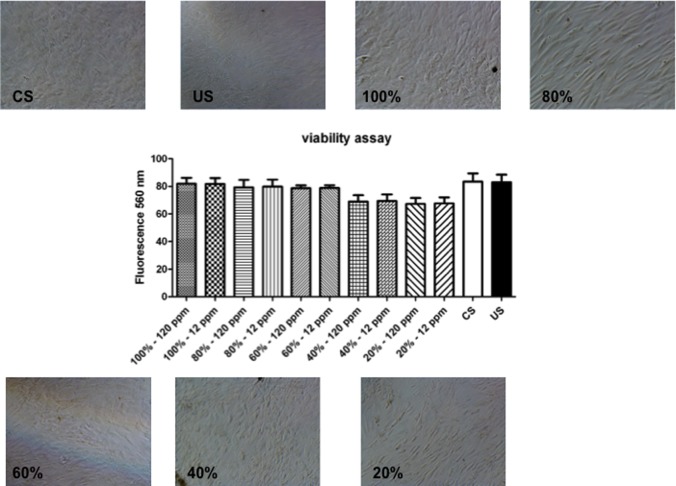
Effect of hemofiltrates on viability and osteogenic differentiation of hMCSs. PrestoBlue assay on cells treated with the five different hemofiltration fractions (20%, 40%, 60%, 80% and 100%), at a final concentrations of 120 or 12 ppm, still ensured suitable cell viability, compared to cell treated with serum from healthy donors (control serum; CS) and serum from uremic patients (uremic serum; US), in normal medium (bar plot). Photomicroscopy images of hMSCs treated with CS, US or in the presence of the five indicated hemofiltrate fractions are also shown.

Also hemofiltrate fractions were tested on MSCs to study the effects on cell viability. Hemofiltrate fractions were diluted in the cell medium to a concentration of indoxyl sulfate (as a typical uremic toxin) in the range of 12–120 ppm, *i*.*e*. above the range of indoxyl sulfate concentration, referred to 10% whole uremic serum (about 2 ppm); see also ref. [53]. Fig. 2 showed that both control and uremic serum provide culture conditions insuring satisfactory cell viability, and addition of hemofiltrate fractions to cell cultures at a final toxin concentration well above that of uremic serum, still insured suitable cell viability for experiments on osteogenic differentiation.

### 2 Cell culture in uremic medium is associated with an alteration of the sRANKL/OPG ratio concentration

**Figure 3:**
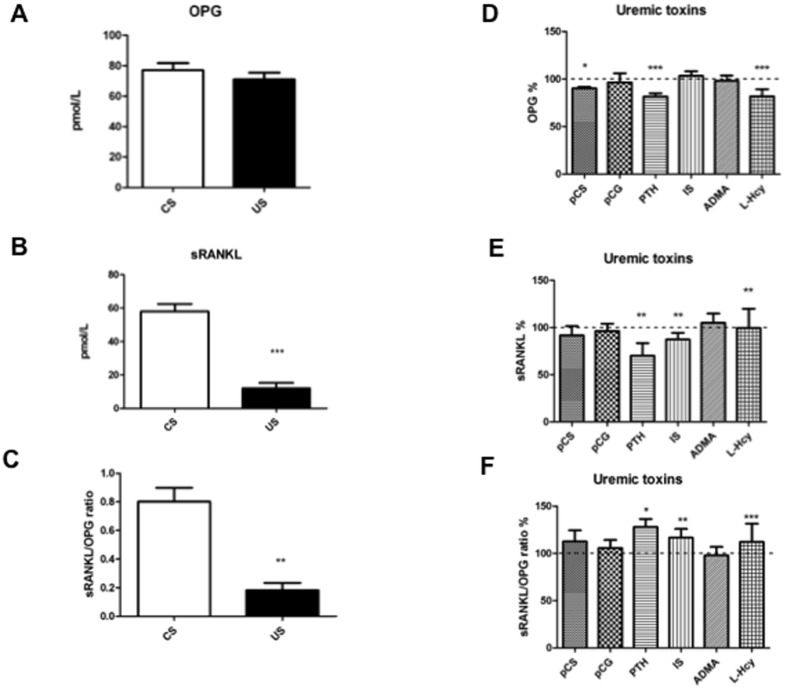
OPG, sRANKL and their ratio in response serum from uremic patients, and the effects of individual uremic toxins. OPG (panel A), sRANKL (panel B) and their ratio (panel C) were measured in hMSC cell medium at 3 days after induction of osteogenic differentiation in the presence of uremic serum (US), compared to control serum (CS). Another set of experiments was performed by inducing osteogenic differentiation in hMSCs cultured in the presence of control serum added with the indicated uremic toxins (pCS, p-cresylsulfate; pCG, p-cresylglucuronide; PTH, parathyroid hormone; IS, indoxyl sulfate; ADMA, asymmetric dimethylarginine; Hcy, homocysteine), compared to control serum alone. Panel D: OPG; panel E: sRANKL; panel F: sRANKL/OPG ratio. *p<0.05, **p≤0.01, ***p<0.001.

A number of key markers of bone metabolism and osteogenic differentiation were monitored according to Fig. 1, after three days from differentiation induction of hMSCs in the presence of uremic serum or selected uremic toxins compared to controls, at concentrations comparable to those detected in uremia. In Fig. 3A, the effects of uremic serum vs. control serum on OPG release in the medium are displayed. Uremic serum did not significantly influence OPG concentration, although there was a trend towards a decrease. Regarding sRANKL (Fig. 3B), uremic serum decreased levels six-fold compared to control serum. The sRANKL/OPG ratio of uremic serum vs. control serum was therefore also markedly decreased (Fig. 3C).

When checking the effects of various uremic toxins on the same markers, pCS, PTH and homocysteine decreased OPG (Fig. 3D). PTH and indoxyl sulfate significantly decreased sRANKL concentration (Fig. 3E). Regarding the sRANKL/OPG ratio (Fig. 3F), PTH and indoxyl sulfate determined an increase.

### 3 The uremic milieu reduces osteogenic differentiation of hMCSs

Markers of bone differentiation, ten days after the start of osteogenic induction (according to Fig. 1), were monitored in the medium of hMSCs cultured in the presence of uremic serum vs. control serum or, in parallel, samples in which selected uremic toxins had been added to control serum vs. no addition samples.

**Figure 4:**
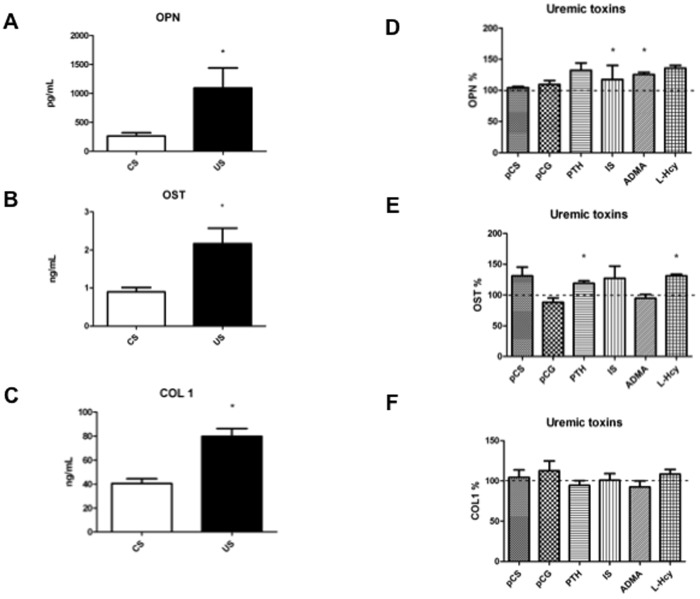
Levels of osteopontin, osteocalcin and collagen type I in response to serum from uremic patients, or individual uremic toxins, during osteogenic differentiation. Osteopontin (OPN, panel A), osteocalcin (OST, panel B) and collagen type I (COL1, panel C) were measured in the medium of hMSCs cultured in the presence of uremic serum (US) vs. control serum (CS). OPN (panel D), OST (panel E) and COL1 (panel F) were also monitored in parallel experiments in which hMSCs were cultured in the presence of control serum added with the indicated uremic toxins (pCS, p-cresylsulfate; pCG, p-cresylglucuronide; PTH, parathyroid hormone; IS, indoxyl sulfate; ADMA, asymmetric dimethylarginine; Hcy, homocysteine) compared to NS as such. *p<0.05.

Uremic serum increased osteopontin (Fig. 4A), osteocalcin (Fig. 4B) and collagen type I (Fig. 4C), in comparison to control serum. This effect was mimicked by ADMA and homocysteine for osteopontin (Fig. 4D), by homocysteine and by PTH for soluble osteocalcin (Fig. 4E). Although a tendency of some tested toxins to influence collagen type I could be detected, no significant changes were observed (Fig. 4F). Alkaline phosphatase expression, a typical late marker of osteogenic differentiation, increased upon induction of osteogenic differentiation both in uremic serum vs. control serum treated hMSC, with no significant difference between the two, nor upon treatment with selected toxins (data not shown).

**Figure 5:**
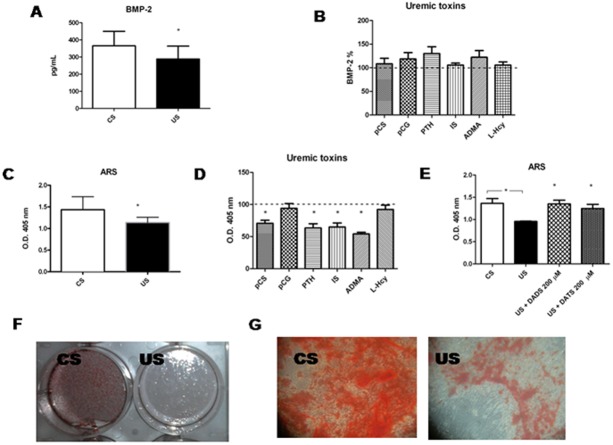
Levels of BMP-2 and ARS in hMSC medium in response to serum from uremic patients or selected uremic toxins and effect of H_2_S donors, during osteogenic differentiation. BMP-2 levels in uremic serum (US) treated hMSCs (panel A) or upon treatment with selected toxins added to control serum (CS) (panel B), and ARS in CS or US treated hMSCs (panel C) or upon treatment with selected toxins (pCS, p-cresylsulfate; pCG, p-cresylglucuronide; PTH, parathyroid hormone; IS, indoxyl sulfate; ADMA, asymmetric dimethylarginine; Hcy, homocysteine) added to NS (panel D) are shown. Effects of H_2_S donors DADS and DATS on ARS in US treated cells (panel E). Examples of ARS stain in hMSCs treated with CS or US, as culture well picture (panel F) and as microscopy images (panel G). *p<0.05.

BMP-2 levels, induced upon osteogenic differentiation, were decreased when hMSCs were treated with uremic serum compared to control serum (Fig. 5A). None of the tested uremic toxins modified BMP-2 (Fig. 5B).

Calcium deposition, as evaluated by the ARS assay, was monitored during late osteogenic differentiation. ARS stain was decreased in the presence of uremic serum, as shown in a representative experiment in Fig. 5F and 5G (red staining was faded in the uremic serum treated cells with respect to control serum) and shown as average levels in Fig. 5C, as well as levels in the presence of pCS, PTH, indoxyl sulfate, and ADMA added to control serum, compared to control serum *per se* (Fig. 5D).

H_2_S donors, DATS and DADS, added to hMSCs, in the presence of uremic serum, significantly increased calcium deposition, monitored as ARS, compared to the H_2_S donor-untreated cells (Fig. 5E). All other osteogenic parameters were not affected by H_2_S donors (data not shown).

### 4 Serum of cinacalcet-treated patients antagonizes the effect of the uremic milieu on hMSC osteogenic differentiation

**Figure 6:**
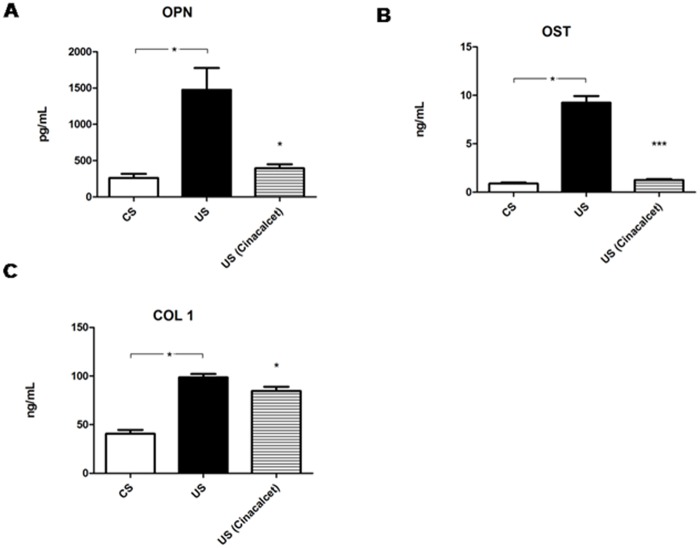
Levels of osteopontin, osteocalcin and collagen type I, in response to uremic serum from patients under cinacalcet treatment. Osteopontin (OPN, panel A), osteocalcin (OST, panel B) and collagen type I (COL1, panel C) during hMSC differentiation in the presence of serum from hemodialysis patients treated with cinacalcet (US cinacalcet), compared to serum from non-treated patients (uremic serum, US). Results from control serum (CS) were added for comparison. *p<0.05, ***p<0.001.

We performed a set of experiments in which hMSCs were induced to differentiation by osteogenic medium in the presence of a pool of sera from hemodialysis patients in therapy with cinacalcet. Serum from cinacalcet-treated uremic patients induced a decrease of osteopontin, osteocalcin, and collagen type I compared to serum from untreated patients. (Fig. 6A, 6B, and 6C). The other parameters were not modified (data not shown).

### 5 Effects of hemodialysis

**Figure 7:**
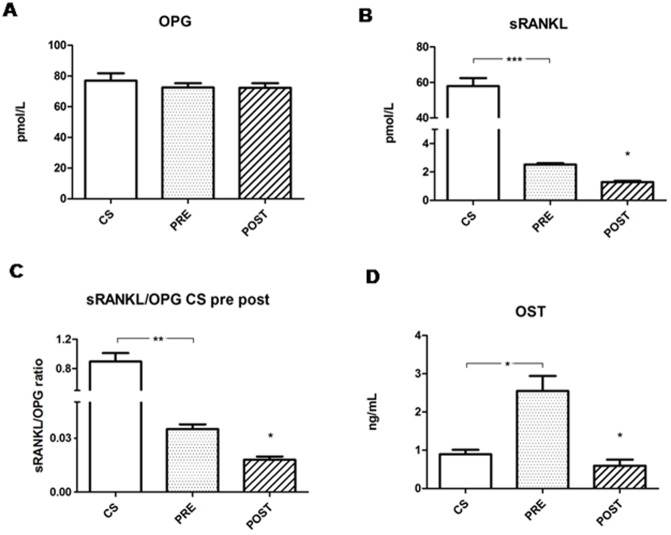
Levels of OPG, sRANKL, sRANKL/OPG, osteocalcin and collagen type I in response to serum from uremic patients after a single hemodialysis session. OPG levels (panel A), sRANKL (panel B) and their relevant ratio (panel C), osteocalcin (OST, panel D) were measured, in the medium, during osteogenic differentiation of hMSC grown in the presence of uremic serum from the same patients before (PRE) and after hemodialysis (POST). Results from control serum (CS) were added for comparison. *p<0.05.

The effect of a hemodialysis session, studied by comparing hMSCs treated with pre-dialysis or post-dialysis serum, was investigated, and we show that a single session did not induce a change in OPG levels (Fig. 7A), while it causes a decrease of sRANKL (Fig. 7B), sRANKL/OPG ratio (Fig. 7C), and osteocalcin (Fig. 7D). All other parameters showed no differences (data not shown).

## Discussion

A uremic environment markedly influences hMSCs, playing a central role in the balance between bone formation and resorption. The reduced bone osteogenesis present in CKD can be explained, at least in part, by the alterations induced by the uremic milieu on hMSCs osteogenic differentiation. Cinacalcet and H_2_S donors, as well as a hemodialysis session, can improve some of these derangements.

CKD-MBD is frequent in CKD and in uremic patients on hemodialysis, in various forms, predominantly adynamic bone disease or renal osteodystrophy [1], due to various determinants [2–3].

HMSCs can tip the balance between bone formation and resorption, by their known capacity to differentiate into osteoblasts, which synthesize the protein components, that allow extracellular osteoid generation, the organic matrix in which mineralization occurs.

Up till now, hMSCs have essentially been used to study calcification occurring in the vascular system, in that the serum from uremic patients (uremic serum) induces a procalcific phenotype, accompanied by matrix remodeling and actual calcification [54]. However, to the best of our knowledge, the effect of uremic milieu on hMSCs differentiation into osteoblasts has never been studied.

Many uremic toxins may be expected to influence hMSCs function and osteogenic differentiation. Several retention compounds in uremia display noxious effects on bone metabolism and function [4–10]. We investigated the effects of the uremic milieu, as well as that of selected uremic toxins (pCS, pCG, PTH, indoxyl sulfate, ADMA, and homocysteine), chosen in consideration of their effects on bone function [4–10].

Compared to serum from healthy control donors (control serum), uremic serum treatment determines several modifications of key regulators of bone remodeling, first of these being a reduction of the ratio sRANKL/OPG, which conforms to what is found *in vivo* in hemodialysis patients [15–17]. The lower ratio sRANKL/OPG is compatible with an initial compensatory response to the uremic environment of the system to favor osteogenesis over osteoclastosis, acting as a limiting factor to excessive resorption.

However, later in the osteogenic differentiation (see Fig. 1), the levels of osteopontin, osteocalcin, collagen type I, are significantly increased under these conditions, while BMP-2, and ARS (a marker of calcium deposition) are decreased, indicating, in the end, that bone formation is reduced in favor of bone resorption.

Osteopontin, a positive modulator of osteoclastosis to prevent excessive bone formation, is increased in cell culture medium upon treatment with uremic serum, consistent with *in vivo* data [18, 21]. The increase of osteopontin *in vitro* during late differentiation opposes the decrease of the sRANKL/OPG ratio (pro-osteogenesis), which is prominent during the first phase of osteogenesis.

Also osteocalcin and collagen type I levels mimic what found *in vivo* [22,23]. This could indicate that they are secreted, and persist in the supernatant in their soluble form, instead of being incorporated into the matrix, thus explaining the reason why *in vitro* calcium deposition is reduced.

On the contrary, BMP-2 levels are lower in our experimental setting, while serum levels are usually higher [55]. BMP-2 is a procalcific protein, and the fact that its levels are lower *in vitro* is in accordance with the hypothesis that the uremic milieu creates an altered microenvironment where bone calcium deposition is reduced. In fact, we also detected lower ARS levels.

All in all, these effects show that, in the balance between bone formation and bone resorption, hMCSs, under uremic medium conditions, uncouple these two processes and are prone towards bone resorption instead of bone formation.

The effects of the selected uremic toxins mimic some of the effects observed in the presence of whole uremic serum. None of the toxins is able to completely replicate the pattern exerted by uremic serum on hMSCs; however, almost all of them reduce ARS levels, and therefore reduce calcification. Regarding sRANKL/OPG, PTH and indoxyl sulfate act in an opposite manner with respect to uremic serum, in that they both increase the ratio. This result is not unexpected for PTH, since this hormone physiologically stimulates bone resorption over deposition. It must be considered that the hMSCs used in our model are exposed to toxins for a limited period of time with respect to cells exposed *in vivo* to the same toxins in the chronic condition of uremia. In addition, it should be noted that many other compounds present in the uremic milieu deserve being tested, aside from the ones we analyzed, for example those that may responsible for the decrease in BMP-2.

Calcimimetics are agents that allosterically increase the sensitivity of the calcium-sensing receptor in the parathyroid gland to calcium [56]. Cinacalcet, the only currently available calcimimetic, is an emerging option in the treatment of secondary hyperparathyroidism in CKD patients on hemodialysis [57–59]. We therefore expect that the serum microenvironment of CKD patients treated *in vivo* with cinacalcet could be more similar to a normal condition, with respect to the alterations of bone metabolism, compared to what was detected in cinacalcet-untreated CKD patients. Results from treatment of hMCSs with cinacalcet-treated patients serum actually showed that the uremic microenvironment was favorably influenced by this *in vivo* treatment, since osteopontin, osteocalcin, and collagen type I behaved more similarly to what was observed in cell cultures grown with control serum. This indicates that therapy with cinacalcet positively modifies the uremic microenvironment with respect to parameters involved in hMSC osteogenic differentiation.

H_2_S, an endogenous vasodilator and antioxidant, is also involved in bone formation and osteoblast proliferation through enhancement of the transcriptional levels of alkaline phosphatase, osteocalcin, and collagen [33], as well as RANKL/OPG [34]. Its levels are low in the plasma of uremic patients on hemodialysis [32], and we have seen *in vitro* experiments that H_2_S treatment decreases the inflammation typical of this condition [60]. It has been already demonstrated that H_2_S regulates bone marrow MSCs in mice [61] and it has been shown that in H_2_S-deficient mice, that display an osteoporotic phenotype, restoring H_2_S via non-toxic donors may provide treatment for diseases such as osteoporosis [61].

Slow-releasing H_2_S donors, such as DADS and DATS, incubated *in vitro* with hMSCs are able to counteract the decrease in ARS levels induced by uremic serum. This finding indicates that these compounds can be of potential therapeutic use, not only with respect to the inflammatory effects of uremia, but also on hMSCs osteogenic differentiation.

Serum from patients after a single hemodialysis session induces a decrease of sRANKL/OPG, because a tendency to a further reduction of sRANKL. Osteocalcin levels were decreased, which is a finding that goes in the opposite direction of what found in cells treated with uremic serum. Therefore, findings are in accordance with the idea that hemodialysis restores the situation in favor of bone formation.

In conclusion, bone modifications in uremia, mediated by secondary hyperparathyroidism and its determinants, can be also influenced by the capability of uremic milieu to alter hMSCs osteogenic potential and differentiation. New therapeutic strategies, such as cinacalcet and H_2_S donors, can be utilized to intervene on these alterations, which need to be further explored. In this respect, the *in vitro* system we set up can be considered as a useful way to study how various conditions and treatments may influence the effects of the uremic microenvironment on hMSC osteogenic differentiation.
